# Effect of storage time on antioxidant activity and inhibition on α‐Amylase and α‐Glucosidase of white tea

**DOI:** 10.1002/fsn3.899

**Published:** 2019-01-25

**Authors:** Ping Xu, Lin Chen, Yuefei Wang

**Affiliations:** ^1^ Department of Tea Science Zhejiang University Hangzhou China; ^2^ Development and Quality Improvement Key Laboratory of Horticultural Plant Growth Chinese Ministry of Agriculture Hangzhou China

**Keywords:** α‐Amylase, α‐Glucosidase, aging, antioxidant activity, polyphenols, white tea

## Abstract

White tea is considered as a special kind of tea not only for its simplest process, but also for its endurable storage. However, little studies have been done about the changes of white tea with increasing aging time, including its composition and health‐imparting effects. In the present work, white tea aged 1 year (WT‐1), 3 years (WT‐3), and 5 years (WT‐5) were collected. Their major chemical compounds, antioxidant activities, and inhibitory effects on α‐Amylase and α‐Glucosidase were evaluated. Results showed that white tea of different storage time showed good antioxidant activity in DPPH, ABTS, and FRAP assay, which decreases with the prolongation of storage time. The inhibitory effects on α‐Amylase and α‐Glucosidase which are key enzymes related to type II diabetes in vitro, are also observed in the similar trend. Meanwhile, prolongation of storage time decreased the content of polyphenols, the main bioactive compounds in tea, which may lead to decrease in the activities investigated.

## 1 INTRODUCTION

Tea is one of the most consumed beverages around the world, second only to water (Damiani, Bacchetti, Padella, Tiano, & Carloni, 2014). It is the processed buds and leaves of the *Camellia sinensis* (L.) plant, which can be generally classified as green, black, white, oolong, dark, and yellow tea with different degrees of oxidation of polyphenols. In Europe and America, white tea is more popular than green tea due to its special flavor (Almajano, Carbó, Jiménez, & Gordon, 2008), and its popularity in China is increasing rapidly. In the processing of white tea, young tea leaves or buds are chosen as raw materials, then spread out to dry in the sun. White Peony (traditionally named Bai Mudan) and Silver Needle (traditionally named Baihao Yinzhen) are the main kind of white tea. The former white tea is produced with a bud and one leaf or two, with a light golden‐brown brewed infusion and a pleasing roasted aroma. Subsequently, it is processed purely from the unopened buds of the plant, with a light yellow infusion.

Many bioactive functions of white tea have been reported in previous research, including cytoprotective effects (Yen et al., 2013), inhibition of de novo lipogenesis (Sohle et al., 2009), improvement of glucose tolerance ability (Islam, 2011), apoptotic effects in non–small‐cell lung cancer cells (Mao et al., 2010), and protection on cutaneous immunity against detrimental effects of UV (Camouse et al., 2009). In some provinces of China, white tea can be used as folk medicine for child flu and diabetes.

White tea can be stored for prolonged periods. Slight fermentation and alteration in chemical constituents is occurring during storage time, leading to distinct flavor and enhanced bioactivities. However, details of compositional changes of white tea at different storage stages remain unclear. Therefore, in the present study, white tea with a storage time of 1 year (WT‐1), 3 years (WT‐3), and 5 years (WT‐5) were chosen. Their major chemical components, antioxidant activities, and inhibitory effects on α‐Amylase and α‐Glucosidase were evaluated.

## 2 MATERIALS AND METHODS

### 2.1 Chemicals

All the catechin standards, 1,1‐diphenyl‐2‐picrylhydrazyl (DPPH), 2,4,6‐tripyridyl‐s‐triazine (TPTZ), 2,2’‐azinobis (3‐ethylbenzothiazoline‐6‐sulfonic acid) diammonium salt (ABTS), porcine pancreatic α‐Amylase (EC 3.2.1.1), and α‐Glucosidase (EC 3.2.1.20) were obtained from Sigma Chemical Corporation(Missouri, USA). Acarbose was purchased from Tokyo Chemical Industry Corporation. (Tokyo, Japan). Other chemicals and reagents used in the experiments were analytical grade or chromatographic grade.

### 2.2 Tea samples and preparation of aqueous infusion

White tea stored for 1 year (WT‐1), 3 years (WT‐3), and 5 years (WT‐5), respectively, were purchased from Fujian Green Leaf Tea Industry Co., Ltd. (Fujian, China). First, 2.50 g white tea samples were soaked in boiled water at 1:50 (w:v, ratio) for 15 min before filtered through filter paper. The same soaking and filtering operations above were repeated again with filter residue. Finally, all filtrates above were merged and distilled water was added to bring the final volume to 250 ml.

### 2.3 Determination of total phenols content (TPC)

Total phenols content in tea infusion was determined according to the Folin‐Ciocalteu method (Shetty, Curtis, & Levin, 1996). 95% ethanol (1 ml), distilled water (5 ml), 50% (v:v) Folin‐Ciocalteu reagent (0.5 ml), and sample infusion (1 ml) were added into test tube and well mixed. Five minutes later, 5% Na_2_CO_3_ (1 ml) was added to previous reaction mixture. Finally, sample mixtures were left for 1 h at room temperature. The absorbance of final reaction mixture was read at 760 nm. Various concentrations of gallic acid standard solution were used to establish the standard calibration curve, and the results of total phenols content were showed as percents of tea sample in dried weight.

### 2.4 Determination of protein content (PC) in tea infusion

The protein content in tea infusion was determined by a slightly revised method of Bradford's (1976). Coomassie Brilliant Blue G‐250, 95% ethanol, and 85% phosphoric acid were used to prepare protein reagent. 1 ml of tea infusion and 5 ml protein reagent were added to the test tube. The absorbance of mixture was measured at 595 nm after 5 min. Bovine serum albumin was used as the standard to be utilized to establish the standard calibration curve, and the results of protein content were showed as percents of tea sample in dried weight.

### 2.5 Determination of free amino acid content (AAC)

Amino acid content was evaluated by nihydrin colorimetry. A sample infusion (1 ml) was added to a volumetric flask (25 ml ‐ capacity), mixed with pH 8.04 phosphate buffer (0.5 ml) and 2% ninhydrin solution (0.5 ml). The total mixture was heated for 15 min in boiling water bath. When cooled at room temperature, distilled water was added to bring it up to 25 ml. Absorbance of reaction mixture was measured at 570 nm. Various concentrations of theanine were used as standard solution to establish the standard calibration curve, and the results were shown as percents of tea sample in dried weight.

### 2.6 Determination of total soluble sugar content (TSSC)

According to the anthrone‐sulphuric acid method (Morris, 1948), total soluble sugar content was determined. Glucose was used as a standard in this method, and the results were shown as percents of tea sample in dried weight.

### 2.7 Determination of catechins and caffeine

The analysis of catechins and caffeine content was performed by HPLC on a SHIMADZU liquid chromatograph with the Absorbance Detector set at 280 nm. The freshly prepared infusions were filtered and injected through a 4.5 μm membrane filter. The solvent compositions included solvent A (acetic acid/acetonitrile/water, 0.5/3/96.5, v:v:v) and solvent B (acetic acid/ acetonitrile /water, 0.5/30/69.5, v:v:v) (Jie et al., 2006). The analysis conditions were as follows: 10 μl of injection volume, TC‐C_18_ 5 μm, 1 ml/min flow rate of solvent compositions. During gradient elution, the solvent B increased linearly from 20% to 65% of the proportion in 35 minutes, and then kept at 20% for a further 5 min. Identification and quantification of catechins and caffeine was according to their retention time and peak area with corresponding standards.

### 2.8 DPPH assay

The scavenging activity of white tea on DPPH free radical was assessed by the method of Ye and Huang (2012) with some adjustment. 0.2 ml of sample infusion previously diluted 10‐fold, or a certain concentration of EGCG (>95%) solution was mixed with 3 ml of 0.1 mM DPPH ethanolic solution. The mixture was incubated in the dark for half an hour at 25°C. Measure the absorbance at 517 nm. The DPPH scavenging activity was calculated using Eq (1).

(1) $$\text{DPPH SA}\left(\%\right)=\left(1-A_{s}/A_{c}\right)\times 100$$

where DPPH SA means DPPH scavenging activity, A_s_ and A_c_ are defined as absorbance of the sample and the control, respectively.

### 2.9 ABTS assay

ABTS assay was performed by Pellegrini's method (Pellegrini, Re, Yang, & Rice‐Evans, 1999). The ABTS cation radical was generated by mixing 7 mM ABTS and 2.45 mM K_2_S_2_O_8_ solution, then incubated in the dark for 12–16 hr. This solution was diluted with distilled water to obtain an absorbance of 0.70 ± 0.02 at 734 nm before being used. 3 ml of ABTS cation radical solution was added to 0.1 ml of sample infusion previously diluted 10‐fold, or a certain concentration of EGCG (>95%) solution, or water as control. Six minutes later, the absorbance of mixture was measured at 734 nm. The ABTS scavenging activity was calculated by Eq (2).

(2) $$\text{ABTS SA}\left(\%\right)=\left(1-A_{s}/A_{c}\right)\times 100$$

where ABTS SA means ABTS scavenging activity, A_s_ and A_c_ are defined as absorbance of the sample and the control, respectively.

### 2.10 Ferric‐reducing antioxidant power (FRAP) assay

The FRAP was carried out according to Benzie and Strain (1999). 10 vol of 300 mM acetate buffer (pH 3.6), 1 vol of TPTZ (10 mM) in HCl (40 mM) and 1 vol of FeCl_3_ (20 mM) were mixed to prepare the working FRAP reagent which would be incubated at 37°C. The absorbance of reagent blank reading was measured at 593 nm. Then, 0.5 ml of sample infusion or a certain concentration of EGCG (>95%) solution was added to 5 ml of the FRAP reagent. 8 min later, a second reading was carried out at the same wavelength. The FRAP value of the sample infusion was determined by subtraction of the initial blank reading with the FRAP reagent alone from the final reading of the FRAP reagent with the sample. A standard curve was made by utilizing various concentrations (50–1000 μM) of FeSO_4_. The ability of ferric‐reducing was expressed as the equivalent to that of 1 μM FeSO_4_.

### 2.11 α‐Amylase inhibition assay

The method of Gonzalez‐Munoz, Quesille‐Villalobos, Fuentealba, Shetty, and Galvez Ranilla (2013) was performed with some modification to measure the α‐Amylase inhibitory activity of the white tea. 250 μl of each infusion sample or a certain concentration of acarbose solution was mixed with 0.02 M sodium phosphate buffer (125 μl, pH 6.9 with 13 U/ml α‐Amylase solution and 6 mM NaCl) and incubated at 25°C for 10 min. After preincubation, 250 μl of a 1% starch solution in 0.02 M pH 6.9 sodium phosphate buffer (containing 6 mM NaCl) was added to test tube at timed intervals. The reaction mixture was then incubated at 25°C for 10 min. The reaction was stopped after adding dinitrosalicylic acid color reagent (1.0 ml). The test tubes were incubated in a boiling water bath for 5 min and cooled to room temperature. Subsequently, the reaction mixture was diluted by adding 7 ml of distilled water and measured at 540 nm. Sample blanks and the control were determined at the same wavelength. Inhibitory activity was presented as inhibition percent and was calculated as in Eq (3).

(3) $$\begin{array}{c} \text{AI}\left(\%\right)=\left[A_{c}-\left(A_{s}-A_{s,b}\right)\right]/\left(A_{c}-A_{b}\right)\times 100 \end{array}$$

where AI means α‐Amylase inhibition, A_s_, A_s.b_ and A_c_ are absorbance of the sample, sample blank (buffer in place of enzyme solution), and the control (buffer instead of sample infusion), respectively.

### 2.12 α‐Glucosidase inhibition assay

The assay for measuring α‐Glucosidase inhibitory activity of the white tea was carried out according to the method of Apostolidis and Lee (2010) with some modification. Sample infusion or acarbose solution (50 μl) was mixed with 0.1 M phosphate buffer (100 μl, pH 6.9 with 1 U/ml α‐Glucosidase). Then the mixture was incubated in 96 well plates at 25°C for 10 min. After preincubation, 0.1 M phosphate buffer solution (50 μl, pH 6.9 with 5 mM pNPG) was added into each well at timed intervals. Subsequently, the reaction mixture was incubated at 25°C for 5 min. Before and after incubation, absorbance was read at 405 nm by microplate reader (Infinite^®^ M200 Pro, TECAN, Switzerland). Buffer instead of sample infusion was utilized as the control. The α‐Glucosidase inhibitory activity was presented as inhibition percent and was calculated as in Eq (4).

(4) $$\text{GI}\left(\%\right)=\left(1-A_{s}/A_{c}\right)\times 100$$

where GI means α‐Glucosidase inhibition, A_s_ and A_c_ are absorbance of the sample and the control, respectively.

### 2.13 Statistical analysis

The results are expressed as mean ± standard deviation (*SD*,* n *= 3), analyzed by analysis of variance (ANOVA) and Tukey's test using SAS system for windows (version 9.1). Pearson correlations were calculated by SPSS 19.0. The results were considered significantly different when *p *< 0.05.

## 3 RESULTS

### 3.1 Chemical composition

The chemical compositions of white tea are presented in Table 1. It can be seen that total soluble sugar content of white tea (TSSC, 3.62%–4.17%) significantly improved with the duration of storage time, while total phenols content (TPC, 17.36%–19.79%), total catechins contents (TCC, 9.40%–12.57%), EGCG content (3.82%– 6.63%), protein content (PC, 3.13%–3.91%), and caffeine content (4.15%–4.27%) showed no difference among samples. There was no linear change trend in amino acid content (AAC, 2.70%–2.96%).

**Table 1:** Chemical analysis of white tea (WT‐1, WT‐3, WT‐5)

Samples	WT‐1	WT‐3	WT‐5
Total phenols (%)	19.79 ± 0.23^a^	18.17 ± 0.04^b^	17.36 ± 0.24^b^
Catechins (%)	12.57 ± 0.08^a^	11.56 ± 0.10^b^	9.40 ± 0.06^c^
EGCG (%)	6.63 ± 0.07^a^	5.01 ± 0.12^b^	3.82 ± 0.08^c^
Protein (%)	3.91 ± 0.09^a^	3.67 ± 0.04^a^	3.13 ± 0.02^b^
Free amino acid (%)	2.96 ± 0.03^a^	2.70 ± 0.05^b^	2.92 ± 0.02^a^
Total soluble sugar (%)	3.62 ± 0.10^b^	3.78 ± 0.07^b^	4.17 ± 0.10^a^
Caffeine (%)	4.20 ± 0.04^a^	4.27 ± 0.05^a^	4.15 ± 0.04^a^

^a‐c^Different letters in the same column mean a difference at significant level (*p* < .05).

### 3.2 Antioxidant activity

**Figure 1:**
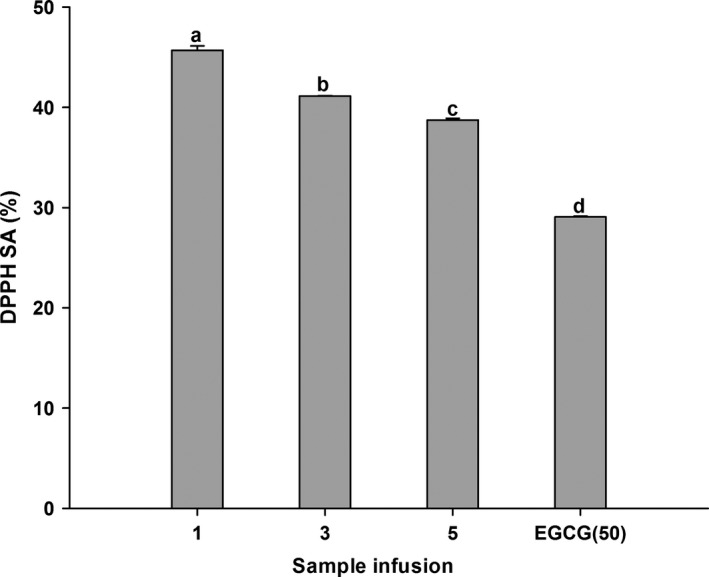
DPPH radical scavenging activity (DPPH SA) of WT‐1, WT‐3, WT‐5, and EGCG. Bars with different letters are significantly different at *p *< 0.05. WT‐1, WT‐3, WT‐5, means white tea had been stored for different years (1, 3, 5)

**Figure 2:**
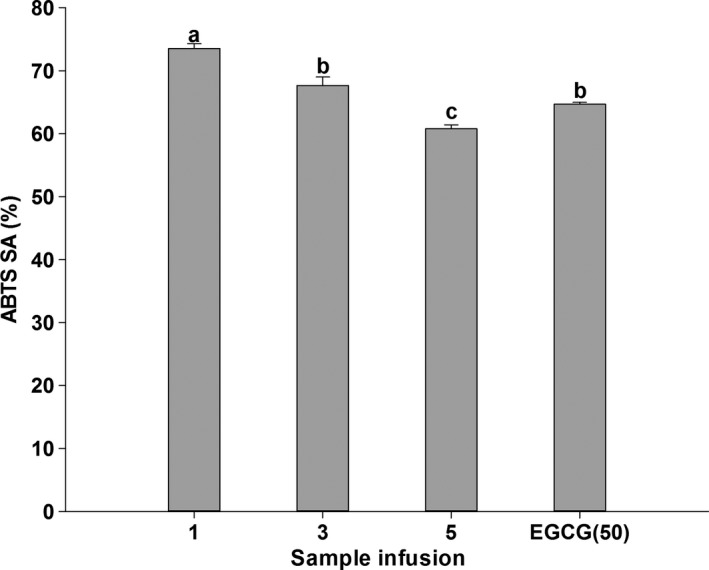
ABTS radical scavenging activity (ABTS SA) of WT‐1, WT‐3, WT‐5, and EGCG. Bars with different letters are significantly different at *p *< 0.05

**Figure 3:**
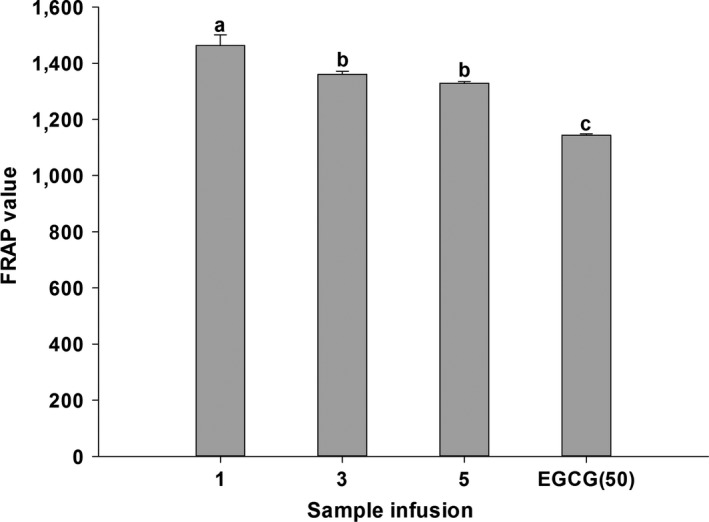
FRAP value of WT‐1, WT‐3, WT‐5, and EGCG. Bars with different letters are significantly different at *p *< 0.05

The results obtained from DPPH assay reported in Figure 1 showed that all sample infusions had significantly stronger DPPH scavenging effects compared to EGCG (50 μg/ml). Furthermore, the highest scavenging activity was observed in WT‐1, lower in WT‐3, and lowest in WT‐5. Similar result can also be seen in ABTS assay (Figure 2). WT‐1 showed significantly better scavenging effects on ABTS than EGCG (50 μg/ml), while WT‐3 possessed no significant difference and WT‐5 exhibited weaker scavenging effects compared to that of EGCG. The antioxidant potential of white tea was also estimated from FRAP assay (Figure 3). WT‐1 showed significantly stronger reducing ability than both WT‐3 and WT‐5, but they all had greater reducing ability than EGCG (50 μg/ml). A significant correlation was found between DPPH assay and TPC (*r* ^2^ = 0.986), TCC (*r* ^2^ = 0.925), EGCG (*r* ^2^ = 0.986), APC (*r* ^2^ = 0.936) in Table 2. The similar correlation was showed between ABTS assay and TPC, EGCG, APC.

**Table 2:** Pearson correlation analysis between some components in tea extracts and their bioactivity

	Total phenols	Catechins	EGCG	Protein	Free amino acids	Total soluble sugar	Caffeine	DPPH	ABTS	FRAP	α‐Amylase inhibition	α‐Glucosidase inhibition
Total phenols	1	0.908^a^	0.979^b^	0.921^b^	0.338	−0.823^a^	0.197	0.986^b^	0.952^b^	0.967^b^	0.875^a^	0.947^b^
Catechins		1	0.949^b^	0.987^b^	−.055	−0.936^b^	0.438	0.925^b^	0.983^b^	0.840^a^	0.647	0.974^b^
EGCG			1	0.936	0.211	−0.871^a^	0.249	0.986^b^	0.969^b^	0.933^b^	0.842^a^	0.988^b^
Aqueous protein				1	0.035	−0.916^a^	0.419	0.936^b^	0.992^b^	0.861^a^	0.649	0.947^b^
Free amino acids					1	0.116	−0.525	0.314	0.117	0.417	0.607	0.067
Total soluble sugar						1	−.195	−0.867^a^	−0.913^a^	−0.831^a^	−0.638	−0.886^a^
Caffeine							1	0.156	0.346	−0.026	−0258	0.365
DPPH								1	0.967^b^	0.966^b^	0.872^a^	0.955^b^
ABTS									1	0.900^a^	0.728	0.968^b^
FRAP										1	0.927^b^	0.877^a^
α‐Amylase Inhibition											1	0.759
α‐Glucosidase Inhibition												1

a *p *< 0.05.

b *p *< 0.01.

### 3.3 Inhibitory effects on α‐Amylase and α‐Glucosidase

**Figure 4:**
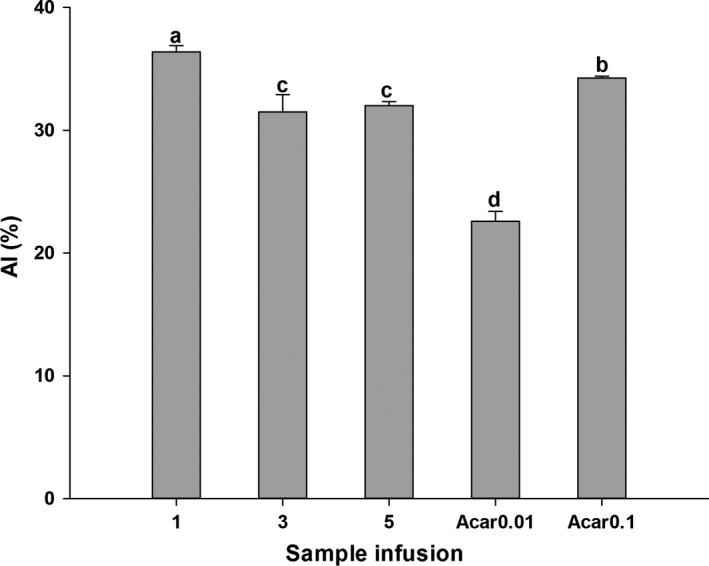
Inhibition of WT‐1, WT‐3, WT‐5, and acarbose on α‐Amylase (AI). Bars with different letters are significantly different at *p *< 0.05

α‐Amylase inhibitory levels are expressed in Figure 4. It can be found that WT‐1 performed better compared to both WT‐3 and WT‐5, when all tested samples had significantly better suppressive effects than acarbose (0.01 g/L). However, WT‐3 and WT‐5 exhibited weaker inhibitory activity than 0.1 g/L of acarbose. So, the inhibitory activity of those white tea samples might have weaker inhibitory activity as storage time prolonged. The correlations between α‐Amylase inhibition and main chemical components of white tea are characterized in Table 2. Obviously, α‐Amylase inhibition had a good correlation with TPC (*r* ^2^ = 0.857) and EGCG (*r* ^2^ = 0.842).

### 3.4 Inhibitory effects on α‐Glucosidase

**Figure 5:**
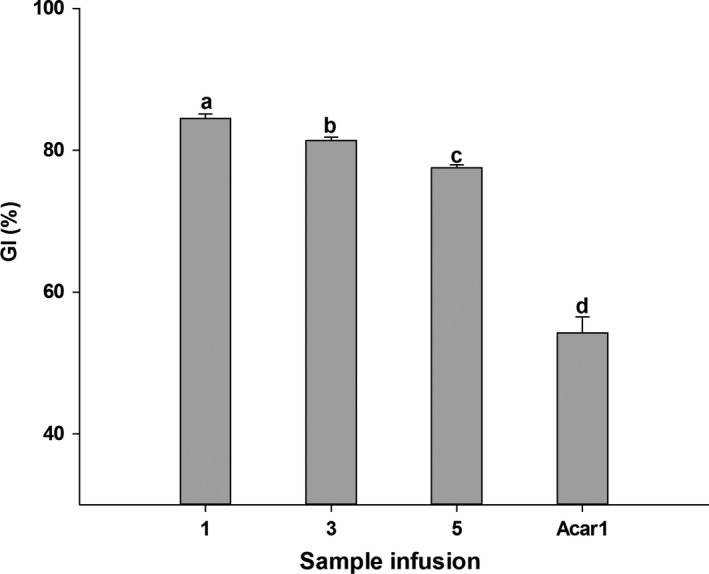
Inhibition of WT‐1, WT‐3, WT‐5, and acarbose on α‐Glucosidase (GI). Bars with different letters are significantly different at *p *< 0.05

The α‐Glucosidase inhibitory activity of white tea is shown in Figure 5. It was interesting to observe that WT‐1, WT‐3, and WT‐5 had excellent suppressive effects on α‐Glucosidase, moreover, significantly better than acarbose (1 g/L). The best performance was seen in WT‐1, while WT‐5 showed it was the weakest. This trend was similar in α‐Amylase inhibition. The correlations between α‐Glucosidase inhibition and the main chemical components of white tea are also shown in Table 2. Apparently, α‐Glucosidase inhibition was well correlated with those compounds (TPC, *r* ^2^ = 0.947; TCC, *r* ^2^ = 0.974; EGCG, *r* ^2^ = 0.988; TPC, *r* ^2^ = 0.947).

## 4 DISCUSSIONS

White tea, processed only through sun withering and drying, is a special category of tea. Contents of chemical components may differ from those in other categories of tea, to some extent. Same as dark tea, white tea can also be stored for prolonged periods with complicated reactions taking place among internal compounds. Jian‐Yong et al. (2011) reported the results of 32.23–33.28 mg/g of total soluble sugar content, 26.36–26.7 mg/g of polysaccharides, 40.33–51.58 mg/g of amino acid content in White Peony (a kind of white tea). The results obtained from the literature showed the tested white tea contained 16.23%–25.95% tea polyphenols, 7.79%–16.25% catechins, 5.2%–9.4% EGCG, and 3.35%–5.74% caffeine (Hilal & Engelhardt, 2007). In comparison with other categories of tea, white tea contains more amino acids and caffeine. Therefore, it is apparent that the results of contents showed in this study were similar to those reports above. Moreover, storage time can also exert great impact on the changes in chemical components contents whose trends are not same. Catechins would automatically oxidize and polymerize when tea are stored. So it was seen in this study that total phenolic content (TPC), total catechin content (TCC), and EGCG significantly decreased when storage time became longer. However, flavonoids content increased as storage time went by, which may due to the structure changes in polyphenols (Shetty et al., 1996). Hydrolysis of protein resulted in loss, which could explain the decreasing trend of protein content (PC) in various storage time. This hydrolysis can also increase free amino acid content, to some degree. However, amino acid itself can undergo oxidation and decomposition. There was no significant change of caffeine content when tea was stored for 5 years in this study, which may result from its relative stability. As a main soluble sugar in tea, tea polysaccharides are always bound with protein and polyphenols. Because of hydrolysis of protein and oxidation of polyphenols as aging time went go, more polysaccharides might be exposed, resulting in the increase. Therefore, total soluble sugar content (TSSC) in white tea may have slight increasing trend in first few years during storage.

Reactive and nitrogen species (RNOS) are produced every moment in human body. It can be safe when RNOS are at a normal level, which owes to the efficient enzymatic and non‐enzymatic defense systems in most living organisms. However, aging and external factors, such as smoking, alcohol, drug, or diet, can impair the ability of such antioxidant protection. Tea is regarded as potent antioxidant agent with its protection against many deleterious diseases (Alarcon, Campos, Edwards, Lissi, & Lopez‐Alarcon, 2008; Almajano et al., 2008). DPPH, ABTS, and FRAP assays, working on different principles, are common methods used for evaluating antioxidant capabilities of substances. Since no single assessment of antioxidant capability is enough (Prior & Cao, 1999), those different assays were all chosen in the present study. The results in this study revealed that white tea had great capacity of scavenging free radicals or possessed excellent antioxidant activities, in accordance with previous studies. Carloni et al. (2013) showed the order of antioxidant profile of various tea: green ≥ low‐caffeine green > white ≥ black Orthodox > black CTC, carried out by ABTS, ORAC and LDL assays. Almajano et al. (2008) observed that white tea had an antioxidant activity comparable to green tea, due to its highest radical‐scavenging activity.

There are small differences in results among DPPH, ABTS, and FRAP assays, but a general trend in antioxidant effects could be as follows: WT‐1 > WT‐3 > WT‐5, which apparently showed that antioxidant effect became weaker with increasing storage time. The results are totally different from the original saying mentioned in folk society. It is commonly known that antioxidant activity is closely related to the various internal bioactive components in tea. Among those constituents, phenolic compounds are considered to be the main and potent antioxidants. There are relatively high concentrations of tea polyphenols in dried white tea. And significantly higher concentrations of total polyphenols, total catechins, caffeine, gallic acid, theobromine, EGCG, EGC, and ECG were observed in white tea, compared to green tea (Hilal & Engelhardt, 2007; Santana‐Rios et al., 2001). As effective antioxidants, they can scavenge superoxide, peroxyl radicals, singlet oxygen, peroxynitrite and hypochlorous acid (Guo et al., 1999; Haenen, Paquay, Korthouwer, & Bast, 1997; Nakagawa & Yokozawa, 2002; Nanjo et al., 1993; Paquay et al., 2000; Scott, Butler, Halliwell, & Aruoma, 1993), due to their structures of A‐ring (hydroxyl groups at the 5 and 7 positions), B‐ring (the ortho‐3’,4’‐dihydroxyl (catechol) group or the 3’,4’,5’‐trihydroxyl (gallate) group) and C‐ring (a gallate group esterified at the 3 position) (Rice‐Evans, Miller, & Paganga, 1996; Wiseman, Balentine, & Frei, 1997). In the present study, significant correlations were found between DPPH (or ABTS, FRAP) assay, and TPC, TCC. Thus, it was not strange that antioxidant activity of white tea became weaker when it was stored for a certain time paralleled with the loss of polyphenols. It was interesting to find that albumin and globulins exhibited the antioxidant activity with scavenging •OH. Antioxidant peptide also showed great scavenging capability to hydroxyl radical and superoxide anion radical in green tea, which may exist in white tea as well.

α‐Amylase and α‐Glucosidase play key roles in carbohydrate digestion. Dietary carbohydrates are degraded into disaccharides depending on α‐Amylase (Satoh, Igarashi, Yamada, Takahashi, & Watanabe, 2015). In the small intestine, those disaccharides (such as maltose and sucrose) are degraded into monosaccharides (such as glucose) by α‐Glucosidase, prior to absorption (Fatmawati, Shimizu, & Kondo, 2011; Oboh, Raddatz, & Henle, 2009). Inhibition on α‐Amylase and α‐Glucosidase to a certain degree can decrease the rate of starch digestion, slowing down the increase of postprandial blood glucose content. So α‐Amylase and α‐Glucosidase are considered as therapeutic targets for modulation of postprandial hyperglycemia in type 2 DM. In the present study, a better inhibitory effect on α‐Amylase was seen in white tea than in acarbose, and these effects decreased with the prolongation of storage time. The same trend was found in the inhibition on α‐Glucosidase. And stronger inhibition on α‐Glucosidase than that on α‐Amylase can also be observed, which indicates that there exists potential strength in white tea with less adverse effects. Because undigested carbohydrate can be fermented in large bowel by bacteria with its inhibition on pancreatic α‐Amylase, this may induce some adverse effects (Samulitis, Goda, Lee, & Koldovsky, 1987). This feature was also reported in black tea (Satoh et al., 2015). Mechanism action of inhibition on α‐Glucosidase may differ from α‐Amylase. The present study implied that polyphenol compounds may possess inhibitory effects on α‐Amylase and α‐Glucosidase, which were also reported in other literatures. Polyphenol‐rich extracts of jute leaf (*Corchorus olitorius*) had good enzyme inhibitory activity on both α‐Amylase and α‐Glucosidase (Oboh et al., 2009). High phenolic‐linked medicinal plants such as Chancapiedra possessed high inhibition on α‐Glucosidase, but their inhibition on α‐Amylase was much lower (Ranilla, Kwon, Apostolidis, & Shetty, 2010). Mixed components possess a stronger inhibition than a single component. EGC showed the best inhibitory effect on α‐Amylase among several monomers (EGCG, ECG, EGC, EC), but was weaker than tea polyphenols extracts. The potential inhibition mechanism was reported by Koh, Wong, Loo, Kasapis, and Huang (2010). Amino acid residue of enzymatic non‐active site would be combined with benzene ring of polyphenol through its hydrogen‐bonding interaction and hydrophobic interaction, thus may reduce the activity of α‐Amylase or α‐Glucosidase. Ademiluyi and Oboh (2013) reported that the antioxidant activity of phenolics may affect the 5 disulfide bridges located on the external surface of amylase, inducing inhibition by modulating changes in the structure of the enzyme. During the storage time, polyphenols, the main components of white tea, got less. So it was natural to observe that inhibitory activity on α‐Amylase or α‐Glucosidase was weaker as time went by. It implied that keeping proper storage conditions was very important for white tea customers, no matter if they would like to preserve it or not.

## 5 CONCLUSIONS

Based on the results obtained, it was concluded that all white tea aged 1, 3, and 5 years possessed potent antioxidant activity and inhibitory effects on key enzymes relevant for type 2 diabetes (α‐Amylase and α‐Glucosidase) in vitro. In addition, extended storage time decreased the content of polyphenols, the main bioactive compounds in tea, which may lead to a decrease on those two enzyme's activities. The inhibitory effects on α‐Amylase and α‐Glucosidase of white tea, especially freshly produced one, provide further experimental evidence for its benefits in diabetes.

## CONFLICT OF INTEREST

We declare that we have no conflict of interest.

## 6 ETHICAL STATEMENTS

The uses of either humans or animals were not applicable in this study.
